# Copeptin Predicts Mortality in Critically Ill Patients

**DOI:** 10.1371/journal.pone.0170436

**Published:** 2017-01-24

**Authors:** Konstantin A. Krychtiuk, Maria C. Honeder, Max Lenz, Gerald Maurer, Johann Wojta, Gottfried Heinz, Kurt Huber, Walter S. Speidl

**Affiliations:** 1 Department of Internal Medicine II—Division of Cardiology, Medical University of Vienna, Vienna, Austria; 2 Ludwig Boltzmann Cluster for Cardiovascular Research, Vienna, Austria; 3 Core Facilities, Medical University of Vienna, Vienna, Austria; 4 3rd Medical Department, Cardiology and Intensive Care Medicine, Wilhelminenhospital Vienna, Austria; 5 Sigmund Freud Private University. Medical School, Vienna, Austria; University of Colorado Denver School of Medicine, UNITED STATES

## Abstract

**Background:**

Critically ill patients admitted to a medical intensive care unit exhibit a high mortality rate irrespective of the cause of admission. Besides its role in fluid and electrolyte balance, vasopressin has been described as a stress hormone. Copeptin, the C-terminal portion of provasopressin mirrors vasopressin levels and has been described as a reliable biomarker for the individual’s stress level and was associated with outcome in various disease entities. The aim of this study was to analyze whether circulating levels of copeptin at ICU admission are associated with 30-day mortality.

**Methods:**

In this single-center prospective observational study including 225 consecutive patients admitted to a tertiary medical ICU at a university hospital, blood was taken at ICU admission and copeptin levels were measured using a commercially available automated sandwich immunofluorescent assay.

**Results:**

Median acute physiology and chronic health evaluation II score was 20 and 30-day mortality was 25%. Median copeptin admission levels were significantly higher in non-survivors as compared with survivors (77.6 IQR 30.7–179.3 pmol/L versus 45.6 IQR 19.6–109.6 pmol/L; p = 0.025). Patients with serum levels of copeptin in the third tertile at admission had a 2.4-fold (95% CI 1.2–4.6; p = 0.01) increased mortality risk as compared to patients in the first tertile. When analyzing patients according to cause of admission, copeptin was only predictive of 30-day mortality in patients admitted due to medical causes as opposed to those admitted after cardiac surgery, as medical patients with levels of copeptin in the highest tertile had a 3.3-fold (95% CI 1.66.8, p = 0.002) risk of dying independent from APACHE II score, primary diagnosis, vasopressor use and need for mechanical ventilation.

**Conclusion:**

Circulating levels of copeptin at ICU admission independently predict 30-day mortality in patients admitted to a medical ICU.

## Introduction

Different pathologies may trigger critical illness in patients requiring admission to an intensive care unit. Despite the fact that the causative underlying conditions are quite heterogeneous and novel technical developments in critical care medicine such as monitoring tools and transient organ replacement were introduced, prognosis remains poor.[1] Reliable biomarkers predicting patient outcome irrespective of the underlying disease are scarce.

Arginine vasopressin (AVP), better known as anti-diuretic hormone (ADH) is an anti-diuretic and vasoconstrictive hormone being released from the posterior pituitary upon various stimuli, such as changes in plasma osmolality and hypovolemia.[2] Additional stimuli include various stressors such as hypoxia, acidosis and severe infections.[3] Besides well-known effects on hemodynamics and osmoregulation, vasopressin constitutes a surrogate for the individuals’ stress level. A short half-life, strong platelet binding capacities and a pronounced instability however, render it unfeasible as a biomarker.[4] Vasopressin is being released together with neurophysin II and copeptin from the prohormone provasopressin.[5] Copeptin, the C-terminal portion of provasopressin, is a stable and simple to measure protein that is being secreted in equimolar amounts to ADH.[6]

Plasma levels of copeptin reliably reflect ADH levels in both healthy and critically ill patients and thus represent a surrogate parameter for precisely that protein.[6, 7] Of note, elevated copeptin levels indicate even moderate stress levels as compared to cortisol for example.[8] In various clinical situations accompanied with elevated endogenous stress levels such as sepsis as well as hemorrhagic and septic shock, copeptin levels were shown to be strongly elevated.[9, 10] Copeptin was shown to exhibit prognostic abilities in various patient cohorts, including exacerbated COPD, decompensated heart failure and myocardial infarction, as well as in patients with stroke.[11–14]

We therefore hypothesized that copeptin, as a reliable marker for the endogenous stress level of the individual patient, might pose a prognostic marker for ICU mortality independent from underlying pathologies. We therefore studied this hypothesis in an unselected cohort of patients admitted to a medical ICU at a tertiary care center.

## Materials and Methods

### Subjects and study design

For this study, designed as a single-center, prospective observational study, we included all consecutive patients that were admitted to the medical ICU of the Department of Internal Medicine II, Medical University of Vienna, between August 2012 and August 2013 that were above the age of 18 years. In addition, we included 34 consecutive stable patients admitted to our cardiology general ward as a control group. Our study was approved by the local ethical committee of the Medical University of Vienna (EK 1101/2012) and complies with the Declaration of Helsinki. To participate in this study, conscious patients had to give written informed consent, while for unconscious patients, the need for informed consent was waived by the Ethical Committee. Our ICU is a tertiary care medical ICU treating the entire spectrum of critically ill medical patients with a focus on acute cardiovascular diseases but also admits patients after undergoing major heart and thoracic surgery. On admission, baseline demographics, cause of admission as well as clinical history, laboratory and vital parameters were recorded. Furthermore, all major interventions preceding ICU admission or taking place within the first 72 hours after ICU admission were noted. This includes need for mechanical ventilation or mechanical assist devices, major surgery, extracorporeal renal replacement, extracorporeal membrane oxygenation and use of catecholamines. For the quantification of disease severity, the simplified acute physiology score II (SAPS II)[15], the acute physiology and chronic health evaluation II (APACHE II)[16] and the sequential organ failure assessment score (SOFA)[17] were used. Mortality within 30 days after ICU admission was noted. In the given time period we included 233 patients, copeptin levels were available for 225 patients. No patients were lost to follow-up.

### Blood sampling

Blood was drawn within 24 hours after admission from the arterial or central venous line. After the initial 3 mL of blood had been discarded, blood was drawn into a serum separator tube and centrifuged at 4°C at 3000 RPM for 15 minutes and stored at -80°C for later analysis. In addition, an EDTA-tube and a 3.8% sodium citrate vacuette tube (all Greiner Bio-One) were collected and treated as described above for later analysis.

### Copeptin measurement

Copeptin was measured using the commercially available CE-certified Copeptin KRYPTOR assay (former B.R.A.H.M.S. Henningsdorf, Germany, now Thermofisher). This kit is an automated sandwich immunoflourescent assay with a detectable range from 0.9 to 500 pmol/L.

### Statistical analysis

Sample size calculation analysis showed that in a cohort with a mortality rate of 25%, given a power of 0.8 and a significance level of 0.01, we would require 200 patients to detect a difference of 50% in copeptin serum levels between non-survivors and survivors.

Categorical variables are summarized as counts and percentages and are compared by the χ2 or by Fisher’s exact test as appropriate. Continuous variables are expressed as median and interquartile range (IQR). Data was compared by Mann-Whitney test. Multiple groups were compared by Kruskal–Wallis one-way analysis of variance and post hoc analysis was performed by Mann-Whitney test adjusted according the method of Bonferroni-Holm. Cox proportional hazard regression analysis was performed to assess the effect of (tertiles of) copeptin on survival. We tested the proportional hazard assumption for all covariates using Schoenfeld residuals (overall test) and the scaled Schoenfeld residuals (variable-by-variable testing). According to the tests the proportional hazards assumption was not violated. Interaction terms were included in the Cox regression model to assess interactions between variables. Kaplan–Meier analysis (log-rank test) was applied to verify the time-dependent discriminative power of quartiles of copeptin.

Two-sided p-values of <0.05 indicated statistical significance. SPSS 18.0 (IBM Corporation, Armonk, NY, USA was used for all analyses.

## Results

### Baseline characteristics

The demographic and clinical characteristics of the 225 patients at baseline are given in Table 1. Median age was 66.7 (54.9–76.7) years and 61% of patients were male. 33% of our patients underwent either cardiac surgery (21%, of those 75% were elective procedures and 25% were emergency surgery; type of surgery comprised CABG in 31.8%, heart valve surgery in 20.5%, combined CABG and heart valve surgery in 22.7% and other procedures in 25% of cases) or heart valve intervention (12%; of those 20% underwent percutaneous edge-to-edge mitral valve repair and 80% transcatheter aortic valve implantation), while of the 67% of patients admitted due to medical reasons, most common causes of admission comprised cardio-pulmonary resuscitation (n = 50; 22%; 37 patients due to primary cardiac arrest, 12 patients due to hypoxia and one patient because of bleeding shock) and heart failure (n = 49; 22%), followed by sepsis (n = 18; 8%) and respiratory failure (n = 16; 7%). Within 30 days after ICU admission, 57 patients (25%) had died.

**Table 1:** Clinical and demographic baseline characteristics of the study population.

	Total n = 225	Survivor (30 days) n = 168	Non-survivor (30 days) n = 57	p-value
**Age (years)**	66.7 (54.9–76.7)	65 (52.7–75.9)	69.6 (59.2–77.5)	0.14
**Male gender, n (%)**	137 (60.9)	100 (59.5)	37 (64.9)	0.53
**Vasopressor use, n (%)**	130 (57.8)	85 (50.6)	45 (78.9)	<0.001
**Mechanical ventilation, n (%)**	130 (57.8)	89 (53)	41 (71.9)	0.013
**Creatinine (mg/dL)**	1.2 (0.9–2.0)	1.1 (0.9–1.7)	1.7 (1.2–2.8)	<0.001
**Creatinine clearance (ml/min)**	45.4 (15.6–90.7)	54.3 (21.7–103.7)	23.4 (6.4–50.8)	<0.001
**Bilirubin (mg/dL)**	0.9 (0.5–1.5)	0.8 (0.5–1.3)	1.1 (0.6–1.8)	0.03
**Lactate (mmol/L)**	1.9 (1.2–3.2)	1.7 (1.2–2.7)	2.8 (1.3–6.7)	0.002
**C-reactive protein (mg/dL)**	3.9 (1.2–10.7)	3.5 (0.9–10.6)	4.8 (2.2–10.9)	0.14
**Procalcitonin (ng/mL)**	0.4 (0.1–1.8)	0.3 (0.1–1.0)	1.3 (0.4–5.0)	<0.001
**Leucocytes (G/L)**	9.2 (7.0–13.5)	8.9 (7.1–13.4)	10.3 (6.5–15)	0.35
**APACHE II score**	20 (12–25)	16 (11–22.5)	27 (23–30)	<0.001
**SAPS II score**	44 (31–57)	38 (29–50)	60 (47–69)	<0.001
**SOFA score**	8 (5–11)	7 (4.5–10)	12 (9–14)	<0.001
**Primary diagnosis**				<0.001
Resuscitation	50 (22)	28 (17)	22 (39)	
Heart failure	49 (22)	33 (20)	16 (28)	
Sepsis	18 (8)	12 (7)	6 (11)	
Respiratory failure	16 (7)	11 (7)	5 (9)	
Cardiac surgery	47 (21)	43 (26)	4 (7)	
Heart valve intervention	26 (12)	25 (15)	1 (2)	
Other	19 (8)	16 (10)	3 (5)	

Numbers are n (%) or median (interquartile range)

### Copeptin levels at admission and primary diagnosis

**Fig 1:**
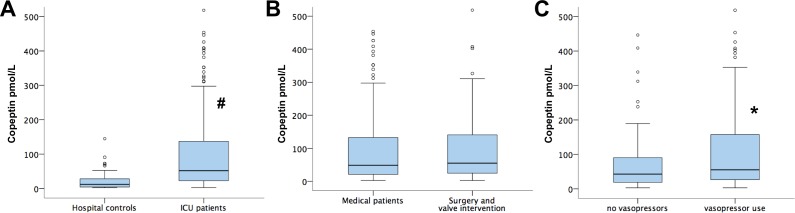
Copeptin serum levels in stable hospital controls, medical and surgical intensive care patients. Serum levels of copeptin in medical control patients compared to patients admitted to an intensive care unit (A); serum levels of copeptin in medical intensive care unit patients as compared to patients who were admitted due to cardiac surgery or heart valve intervention (B); patients that needed vasopressors show increased levels of copeptin as compared to patients without vasopressor use (C); outliers not shown; # p<0.0001; * p<0.05

Median copeptin levels at admission were 51.5 IQR 22.6–137.4 pmol/L, thus much higher than in our medical control group (n = 35) that exhibited median copeptin levels of 12 IQR 4.3–32.4 pmol/L; p<0.0001 for ICU patients versus control patients, respectively; Fig 1A). Copeptin levels were not different in patients admitted after cardiac surgery or heart valve surgery as compared to patients admitted due to medical critical illness (55.2 IQR 23.8–144.2 pmol/L versus 48.9 IQR 21–132.6 pmol/L; p = 0.54; Fig 1B.) Within the group of medical patients, highest admission levels were found in patients with cardiogenic shock (133.8 IQR 29.9–294.9 pmol/L), followed by the group of patients with sepsis or septic shock (96.1 IQR 27.9–124.1 and 109.4 IQR 35.9–144.7 pmol/L, respectively). Copeptin admission levels showed a weak correlation with the disease severity markers APACHE II (R = 0.21, p = 0.002) SAPS II (R = 0.21, p = 0.001) and SOFA-Score (R = 0.18, p = 0.006), respectively. When analyzing medical and surgical patients individually, significant correlations were found between copeptin levels and APACHE II and SOFA-score in both subgroups, while the correlation between SAPSII and copeptin levels was significant in medical patients, in surgical patients, the association did not reach statistical significance (data not shown). In contrast, there was no association between copeptin levels and use of mechanical ventilation (p = 0.81) while patients who required catecholamine support showed higher copeptin levels as compared to those who did not (55.3 IQR 26.2–158.5 pmol/L versus 42.8 IQR 18.5–95.1 pmol/L; p = 0.029; Fig 1C). There was no association between copeptin levels and systolic blood pressure or mean arterial blood pressure in the whole population, as well as in those patients not receiving catecholamines. Interestingly, copeptin and NT-proBNP admission levels were significantly correlated (R = 0.37, p<0.001).

### Copeptin levels and mortality

**Fig 2:**
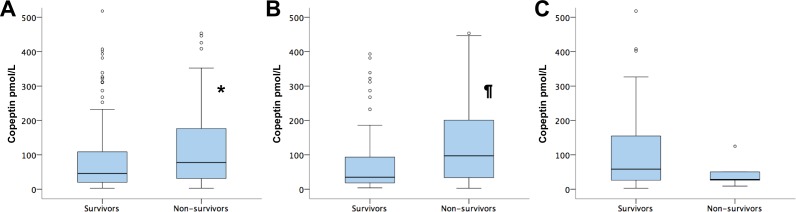
Copeptin serum levels and 30 day survival. Serum levels of copeptin in 30-day survivors and non-survivors in the total cohort (A), in medical patients (B) and in patients that were admitted because of cardiac surgery or heart valve intervention (C); outliers not shown; * p<0.05; ¶ p<0.005

**Fig 3:**
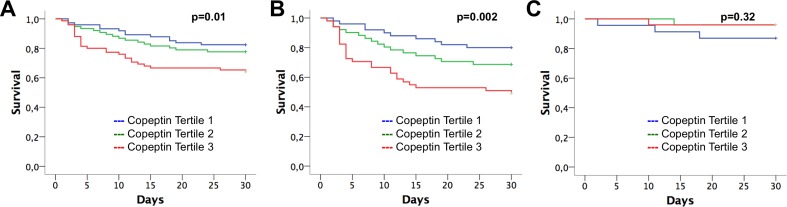
Survival according to tertiles of copeptin. Survival according to tertiles of copeptin in the total cohort (A), in medical patients (B) and in patients that were admitted due to cardiac surgery or heart valve intervention (C);

Patients that died within 30 days after ICU admission had significantly higher copeptin levels as compared to patients that survived (77.6 IQR 30.7–179.3 pmol/L versus 45.6 IQR 19.6–109.6 pmol/L; p = 0.025; Fig 2A). Patients with serum levels of copeptin in the third tertile at admission had a 2.4-fold (95% CI 1.2–4.6; p = 0.01) increased mortality risk as compared to patients in the first tertile (Fig 3A).

### Copeptin predicts mortality in medical but not in surgical patients

In medical patients (n = 152), copeptin was significantly higher in non-survivors as compared to 30 day-survivors (97.2 IQR 33.4–210.3 pmol/L versus 34.9 IQR 18.3–94.3 pmol/L; p = 0.002; Fig 2B). In contrast, in patients after heart valve intervention or heart surgery (n = 73), copeptin was not associated with survival (28.3 IQR 17.5–87.7 pmol/L versus 58.4 IQR 25.5–155.2 pmol/L; p = 0.24; Fig 2C). Medical patients with serum levels of copeptin in the third tertile had a 3.3-fold (95% CI 1.6–6.8; p = 0.002) increased mortality risk as compared to patients in the first tertile (Fig 3B) whereas tertiles of copeptin were not associated with survival in surgical patients (Fig 3C). In medical patients, the third tertile of copeptin predicted risk independent from APACHE II score, primary diagnosis, vasopressor use and need for mechanical ventilation (Table 2).

**Table 2:** Multivariate analyses of the association between elevated copeptin levels and 30-day mortality in medical patients (n = 152).

**Unadjusted**			
	**Hazard ratio**	**95% CI**	**p-value**
**First tertile (2.1–29.7 pmol/L)**	1		
**Second tertile (29.7–91.9 pmol/L)**	1.7	0.8–3.8	0.19
**Third tertile (95.1–710.0 pmol/L)**	3.3	1.6–6.8	0.002
**Adjusted for age, gender, APACHEII score, vasopressor use, mechanical ventilation and primary diagnosis**			
**First tertile (2.1–29.7 pmol/L)**	1		
**Second tertile (29.7–91.9 pmol/L)**	1.5	0.6–3.4	0.38
**Third tertile (95.1–710.0 pmol/L)**	2.5	1.1–5.4	0.024

## Discussion

In this single-center, prospective observational study including 225 critically ill patients admitted to a medical ICU, patients who died within 30 days after ICU admittance showed significantly higher circulating copeptin levels as compared to those who survived. When stratified according to tertiles of circulating copeptin levels, patients in the highest tertile exhibited a 2.4-fold increased risk of dying within 30 days when compared to the patients in the first tertile.

One third of our patient population consisted of patients admitted due to cardiac surgery or interventional heart valve intervention, while the medical patients were primarily admitted after cardio-pulmonary resuscitation, cardiogenic shock or acute heart failure and sepsis. Of interest, copeptin admission levels did not differ between those two patient groups. When evaluating the prognostic abilities of admission copeptin levels in medical and surgical patients, copeptin exhibited no prognostic abilities in those patients admitted due to cardiac surgery or heart valve intervention. However, in the medical group (n = 152), patients in the highest tertile of copeptin exhibited a 3.3-fold increased risk of dying within 30 days as compared to patients in the lowest tertile. Most importantly, these findings were independent from APACHE II score, admission cause, use of vasopressors and need for mechanical ventilation. In addition, we recruited 35 patients admitted to the general cardiology ward of our department. These patients showed markedly lower copeptin levels as compared to the ICU patients, thus supporting the notion of copeptin as a marker for endogenous stress in critically ill patients.

When further evaluating admission causes within the medical subgroup we could demonstrate the highest levels of copeptin in patients with cardiogenic shock, followed by patients with sepsis or septic shock. Regarding sepsis, the copeptin values obtained in our study are consistent with previously published observations including a patient cohort of 101 critically ill patients with sepsis, severe sepsis and septic shock.[10] Another study evaluating 461 patients admitted to the emergency department of a tertiary university hospital with signs of sepsis, demonstrated predictive abilities of copeptin levels at admission for the occurrence of septic shock and mortality.[18] In a small study evaluating 41 septic patients a strong correlation between copeptin and the disease severity score APACHE II was demonstrated.[19] In our cohort, only weak correlations between copeptin and APACHE II scores, as well as other established scores including the SAPS II and the SOFA score were demonstrated. Considering acute heart failure, several studies demonstrated elevated levels of copeptin being predictive of mortality, such as a substudy of the BACH study, including 557 patients with AHF.[20] A substudy of the OPTIMAAL trial including 221 patients with heart failure after acute myocardial infarction suggested prognostic abilities of copeptin in predicting mortality and a composite cardiovascular endpoint.[21] However, in contrast to our study, no patients with cardiogenic shock were included and the observed copeptin values were, as one would expect, markedly lower as compared to our cohort of cardiogenic shock patients.

Biological stress is the organism’s reaction towards a stressor that threatens to derail the body’s homeostatic balance, such as an acute critical illness. Activation of the hypothalamic-pituitary-adrenal (HPA) axis via brain stem and limbic pathways induces the release of corticotropin releasing hormone (CRH) from the hypothalamus, which in turn stimulates the release of adrenocorticotrophic hormone (ACTH) from the pituitary gland. Another hypothalamic hormone produced during stress is AVP, which is being released together with copeptin from a precursor protein. AVP seems to exhibit potentiating actions on the CRH-induced ACTH release, which in turn induces cortisol release from the adrenal cortex and represents a major component in the general answer to such a stressor.[22–24] In addition, hypotension, common in shock states, is another stimulus for AVP secretion to stimulate fluid retention to increase blood pressure in shock states, while additional vasoconstrictive effects are mediated via AVP in higher concentrations.

We therefore postulate that due to the dual stimulation of AVP release in shock states, via endogenous stress and hypotension, we demonstrate highest levels of copeptin in cardiogenic and septic shock patients. However, it has to be noted that no association between systolic or mean arterial blood pressure and copeptin levels could be found. When interpreting blood pressure findings in this study it has to be acknowledged that more than 50% of patients received catecholamine support upon study inclusion and blood sampling, making blood pressure levels not reliable thus hindering testing this hypothesis. However we could observe significantly higher copeptin levels in those patients receiving catecholamine support as well as a significant correlation between copeptin and NT-proBNP admission levels. The latter two findings may indirectly support our hypothesis.

However, cardiogenic and septic shock patients comprised only a subset of patients within our study, the biggest groups among medical patients were patients admitted after cardio-pulmonary resuscitation, acute heart failure, sepsis and respiratory failure. There is to the best of our knowledge only one study evaluating copeptin levels in comatose patients after CPR including 134 patients that could demonstrate an association of elevated copeptin levels and cerebral functional outcome without evaluating mortality as an outcome parameter.[25]

We believe that the heterogeneity of the study population in terms of admission causes, reflecting a typical critically ill population that critical care physicians face on a day-to-day basis, represents a major strength of our data, representing a large cross-sectional study including 225 critically ill patients in which we could demonstrate that copeptin admission levels are associated with outcome irrespective of the underlying disease. Our interpretation of the data presented here is that copeptin admission levels represent the overall endogenous stress of the patient reflecting disease severity as a surrogate parameter thereby predicting mortality, independent of the underlying pathology. We want to point out that we also included primary diagnosis into our multivariate analysis and could demonstrate that the observed predictive abilities were independent from admission cause.

A few limitations of the herein presented study need to be mentioned. First, this was a single-center study including patients from only one single medical intensive care unit. However, the nature of an all-comer study with a vast heterogeneity of underlying pathologies ranging from cardiogenic shock and cardiac arrest to sepsis and admission after cardiac surgery or heart valve intervention demonstrates the strength of our study. As this was an observational study, we can only evaluate and calculate predictive abilities of copeptin admission levels without drawing any definitive functional conclusions of this association. Accordingly, the presented results may possibly be explained by unmeasured confounding factors, for which reason we tried to adjust for baseline imbalances using multivariable modeling. However, we admit that the possibility of undetected or residual confounding cannot be ruled out. Furthermore, as we only measured copeptin levels on admission we cannot rule-out whether measuring copeptin at a later time point or time-dependent changes serve as better mortality predictors.

In conclusion, we herewith provide evidence for the predictive abilities of copeptin measurements at ICU admission for 30-day survival of unselected critically ill patients admitted to a cardiovascular medical ICU. These findings were independent from APACHE II score, cause of admission as well as need for vasopressor use and mechanical ventilation. Thus, our findings provide novel evidence for plasma copeptin levels as an independent predictor for mortality in critically ill patients requiring ICU admission. These results should be confirmed with an external validation cohort and could further stimulate research regarding the use of copeptin as a novel risk marker in critical ill patients.
